# Early Exposure to Neonatal Neurology and Cranial Ultrasound: A Pilot Evaluation of an Undergraduate Course

**DOI:** 10.3390/pediatric18050124

**Published:** 2026-09-22

**Authors:** Andrea Calandrino, Samuele Caruggi, Mariya Malova, Sara Uccella, Francesco Vinci, Paolo Massirio, Marcella Battaglini, Alessia Pepe, Alessandro Parodi, Luca Antonio Ramenghi

**Affiliations:** 1 Neonatal Intensive Care Unit, Department of Maternal and Neonatal Health, IRCCS Istituto Giannina Gaslini, 16147 Genoa, Italy; andrea.calandrino@edu.unige.it (A.C.); francescovinci@gaslini.org (F.V.); paolomassirio@gaslini.org (P.M.); marcellabattaglini@gaslini.org (M.B.); alessiapepe@gaslini.org (A.P.); alessandroparodi@gaslini.org (A.P.); lucaramenghi@gaslini.org (L.A.R.); 2 Child Neuropsichiatry Unit, Department of Pediatric Sciences, IRCCS Istituto Giannina Gaslini, 16147 Genoa, Italy; sara.uccella@unige.it; 3 Department of Neuroscience, Rehabilitation, Ophthalmology, Genetics and Mother/Child Sciences (DINOGMI), University of Genova, 16132 Genoa, Italy

**Keywords:** neonatal neurology, cranial ultrasound, undergraduate medical education, medical students, clinical training, neurodevelopment, educational intervention, bedside teaching

## Abstract

**Background/Objectives:** Undergraduate medical exposure to neonatal neurology and cranial ultrasound remains limited, despite their relevance to neonatal clinical care. We developed a two-day monographic course integrating developmental neuroscience, neonatal clinical reasoning, neurological assessment, and bedside-oriented cranial ultrasound. This pilot study aimed to evaluate the acceptability, perceived educational relevance, and self-reported learning associated with the intervention. **Methods:** We conducted a single-center cross-sectional anonymous post-course evaluation among fourth- to sixth-year medical students at the University of Genoa, Italy, in October 2025. Seventeen students attended the course, and all completed an anonymous post-course questionnaire (response rate, 100%). The locally developed questionnaire included demographic items, five-point Likert-scale questions, categorical responses, retrospective self-ratings of knowledge before and after course attendance, and optional free-text comments. Descriptive statistics and 95% confidence intervals were calculated. Paired comparisons were performed using the Wilcoxon signed-rank test. **Results:** Seventeen students completed the questionnaire. The mean overall course evaluation was 4.18 ± 1.19/5 (95% CI 3.57–4.79) with a median rating of 5 (IQR 3–5), while perceived curricular relevance was rated 4.94 ± 0.24/5. The practical component received higher ratings than the theoretical component (4.88 ± 0.33 vs. 4.47 ± 0.62; median 5 [IQR 5–5] vs. 5 [IQR 4–5]; exploratory Wilcoxon signed-rank *p* = 0.020). Retrospective self-rated knowledge increased from 3.29 ± 1.10 before the course to 4.12 ± 0.70 after course completion (median 4 [IQR 4–5] vs. 3 [IQR 2–4]; exploratory *p* = 0.010). All respondents considered the educational format potentially applicable to other areas of the medical curriculum and 14/17 supported its replication within it. Eight respondents (47.1%) provided at least one optional free-text comment. Positive feedback highlighted the integration of theoretical and practical teaching and the small-group format, while suggestions included closer temporal integration of theory and practice (n = 3) and additional opportunities for direct hands-on cranial ultrasound experience (n = 2). **Conclusions:** This pilot course was highly acceptable and was associated with higher post-course knowledge self-ratings than retrospective pre-course ones. The practical cranial ultrasound component was particularly well received. A compact theory-to-practice format may offer an educational approach that was well accepted and perceived as valuable by participating students to introducing complex and underrepresented neonatal topics during undergraduate medical education. However, future multicenter studies incorporating objective learning outcomes and longer-term follow-up are needed.

## 1. Introduction

Neonatal neurology is one of the clinical areas in which early human development becomes most directly visible at the bedside [1]. Neurodevelopment, prematurity-related brain vulnerability, neurological disorders of the term neonate, and the interpretation of early neurological signs all require learners to connect developmental biology with clinical reasoning [2]. Despite the central role of developmental neurology in perinatal and neonatal medicine, undergraduate exposure to neonatal neurology remains limited, particularly in highly specialized areas and in bedside imaging applications [3,4].

This gap should also be viewed in the context of a broader educational challenge: neurology continues to be perceived by many students as difficult, abstract, and insufficiently integrated with clinical medicine [3].

In parallel, pediatric and neonatal education is increasingly moving toward structured, experiential, and clinically anchored teaching strategies [5]. This is particularly important when the educational goal includes not only conceptual understanding, but also the acquisition of an initial bedside-oriented skill. In neonatal medicine, cranial ultrasound occupies a distinctive position because it is both a practical bedside imaging tool and an entry point into the pathophysiology of preterm and term brain injury [6]. However, formal exposure to neonatal cranial ultrasound usually occurs during postgraduate rather than undergraduate training [4].

Against this background, we designed a two-day monographic course for medical students that combined neonatal neurology teaching with practical exposure to neonatal cranial ultrasound. The rationale was twofold: first, to make a cognitively demanding field more accessible by grounding it in developmental and clinical reasoning; second, to expose students early to a bedside imaging modality that is highly relevant to neonatal brain-oriented care. The aim of this pilot study was to describe the acceptability and perceived educational value of a two-day undergraduate course integrating neonatal neurology and cranial ultrasound.

## 2. Materials and Methods

### 2.1. Study Design and Setting

We conducted a single-center pilot descriptive cross-sectional post-course educational quality evaluation of a two-day extracurricular undergraduate course in neonatal neurology and cranial ultrasound. The course was delivered to fourth- to sixth-year medical students at the University of Genoa, Genoa, Italy, in October 2025. The evaluation was undertaken to assess students’ perceived quality, acceptability, educational relevance, and perceptions of the educational experience, and to obtain feedback for future improvement of the course.

### 2.2. Participants

The course was proposed by the Director of the Medical School as an extracurricular practical educational activity complementary to the standard didactic curriculum. Through an institutional course-selection format, medical students from the fourth to the sixth year were invited to express their willingness to participate. A total of 17 students requested to attend the course, and all participants were subsequently invited to complete an anonymous questionnaire immediately after the course. The post-course evaluation was voluntary and was administered electronically using Microsoft Forms through an institutional University of Genoa Microsoft 365 account. The questionnaire was configured so that respondents’ names, institutional e-mail addresses, and other directly identifying information were not recorded. Participation in the questionnaire had no effect on students’ academic assessment, course participation, or subsequent educational activities. All 17 students completed the questionnaire, corresponding to a response rate of 100%. Survey participation was voluntary, and no written consent form was collected from student respondents. No directly identifying information was collected through the questionnaire.

Among the 17 participants, 5 were fourth-year, 6 were fifth-year, and 6 were sixth-year medical students. The course attracted students with an expressed interest in pediatrics and, in particular, neonatal medicine. None of the participants had previous experience with neonatal cranial ultrasonography, making this their first exposure to the technique.

### 2.3. Course Structure

The course was delivered over two consecutive days and combined didactic teaching with practical, bedside-oriented learning. The main learning objectives were to provide students with an understanding of the clinical role of neonatal cranial ultrasound in the assessment of neurological conditions and to help them recognize when the technique may be useful based on clinical findings or relevant neonatal history. Particular emphasis was placed on the potential of cranial ultrasound as a bedside imaging technique, highlighting its rapidity, accessibility, portability, non-invasive nature, absence of ionizing radiation, and ability to provide clinically relevant information at the bedside. The course was not intended to confer independent neurological examination or cranial ultrasound scanning competence. Rather, students were expected to understand the clinical indications and potential applications of the technique and its role within the diagnostic evaluation of the neonate.

On the first day, students attended 4.5 h of theoretical teaching in the morning, followed by a lunch break and a further 3 h of teaching in the afternoon. The theoretical sessions covered neonatal brain development, brain vulnerability in preterm infants, neurological disorders in term neonates, and the principles of neonatal neuroimaging. The cranial ultrasound component included an introduction to the main anatomical structures visualized through standard coronal and sagittal planes, the principal pathological conditions that can be identified using the technique, and the role of magnetic resonance imaging as a complementary modality for further diagnostic assessment. Teaching was delivered through frontal lectures supported by PowerPoint presentations and educational videos by an experienced neonatologist from the Neonatal Pathology Unit of the Istituto Giannina Gaslini.

On the second day, students participated in 5 h of activities in the morning and a further 2 h in the afternoon. The neurological assessment activity consisted of a structured explanation of the neonatal neurological examination, supported by video demonstrations.

The cranial ultrasound activity included an initial theoretical explanation of the use of a micro-convex transducer through the anterior fontanelle, with demonstration of the main standard coronal and sagittal imaging planes, relevant anatomical structures, and principal pathological findings using illustrative images and educational videos. This was followed by supervised hands-on sessions in small groups. The 17 students were divided into three groups of six, six, and five students, respectively, with one neonatologist tutor from the Istituto Giannina Gaslini assigned to each group, corresponding to tutor-to-student ratios of 1:6 and 1:5.

Bedside cranial ultrasound examinations were performed in three healthy term neonates admitted to the neonatal care unit (nursery) of the Istituto Giannina Gaslini. The neonates were selected because cranial ultrasound was clinically indicated as part of routine care before discharge. Written informed parental consent was obtained after the parents had been informed about the clinical indication for the examination and its use for educational purposes.

During each session, the ultrasound examination was initially performed by the tutor. Subsequently, each student had the opportunity, under direct supervision, to handle the transducer and attempt to reproduce the main coronal and sagittal imaging planes previously demonstrated. During the practical activity, students were also asked to identify the main anatomical structures presented during the theoretical session. The activity was intended to provide supervised familiarity with probe handling, standard imaging planes, and the correlation between ultrasound findings and the clinical context, rather than to assess or establish independent scanning competence. The timetable and delivery methods are summarized in Table 1. The neonatal examinations were part of the supervised educational activity and were not research procedures. No neonatal clinical, imaging, or outcome data were collected, extracted, or analyzed for the post-course educational evaluation reported in the present manuscript.

### 2.4. Survey Instrument

At the end of the course, participants were invited to complete an anonymous questionnaire developed specifically for the local evaluation of the educational activity and for course quality improvement. The questionnaire was administered electronically using Microsoft Forms through the University of Genoa institutional Microsoft 365 environment. Participation was voluntary, and no directly identifying information was collected.

The questionnaire assessed students’ perceived quality and acceptability of the course, perceived curricular and professional relevance, clarity of teaching, perceived usefulness of the theoretical and practical components, retrospective self-ratings of knowledge before and after course attendance, interest in further training, and suggestions for future improvement. Optional free-text fields were also included. The questionnaire was developed as a local educational evaluation instrument and had not undergone formal psychometric validation. The complete questionnaire, including response anchors and free-text prompts, is provided as Supplementary File S1.

### 2.5. Outcomes

The primary outcomes were overall course acceptability, perceived relevance of the theoretical and practical components, and the change in retrospective self-rated knowledge from before to after course attendance. Secondary outcomes included students’ perceptions of the transferability of the educational format to other curricular areas, participants’ perceived acquisition of an introductory professionally relevant competence, the extent to which the course met or exceeded expectations, support for curricular replication, and its possible influence on consideration of pediatrics or neonatology as a future career path. Optional free-text responses were examined descriptively to provide additional context for the quantitative findings and to identify recurring suggestions for future course development. No formal qualitative analysis was performed.

### 2.6. Statistical Analysis

Likert-scale responses were summarized using means and standard deviations (SDs), 95% confidence intervals (CIs), medians and interquartile ranges (IQRs), and the distribution of responses across the original five response categories. Categorical variables are reported as counts and percentages using the original response categories. Exploratory paired comparisons between ratings of the theoretical and practical components and between retrospective pre-course and post-course knowledge self-ratings were performed using the Wilcoxon signed-rank test. Given the pilot descriptive nature of the study and the small sample size, these comparisons were considered exploratory and were not interpreted as estimates of educational effect.

## 3. Results

### 3.1. Participant Characteristics

A total of 17 students attended the course, and all 17 completed the post-course questionnaire, corresponding to a response rate of 100%. Participants were in the fourth (n = 5), fifth (n = 6), or sixth (n = 6) year of the medical degree program. Thirteen respondents were female (76.5%) and four were male (23.5%). The course attracted students with an expressed interest in pediatrics, particularly neonatal medicine, and none had previous experience with neonatal cranial ultrasonography. Baseline specialty interest was not formally assessed using a pre-course questionnaire.

Likert-scale items were scored from 1 to 5 and are reported using descriptive statistics, including means and standard deviations (SDs), 95% confidence intervals (CIs), medians and interquartile ranges (IQRs), and the distribution across response categories, as appropriate (Table 2 and Table 3). Categorical items are reported according to their original response options. No objective assessment of knowledge acquisition or clinical or ultrasound performance was conducted.

### 3.2. Course Acceptability and Perceived Educational Value

Overall, participants reported positive perceptions of the course, with ratings predominantly distributed in the upper response categories (Table 3). The course received a mean overall evaluation of 4.18 ± 1.19 on a five-point Likert scale (95% CI 3.57–4.79), with a median rating of 5 (IQR 3–5). Ten participants (58.8%) assigned the highest rating of 5, two (11.8%) assigned a rating of 4, four (23.5%) a rating of 3, and one (5.9%) a rating of 1.

Perceived alignment of the course content with the existing medical curriculum was rated 4.94 ± 0.24 (95% CI 4.82–5.07), with a median of 5 (IQR 5–5). Interest generated by the course topics received a mean rating of 4.65 ± 0.61 (95% CI 4.34–4.96), with a median of 5 (IQR 4–5).

Students rated the clarity of the topics presented at 4.76 ± 0.56 (95% CI 4.48–5.05), with a median of 5 (IQR 5–5), while the clarity of faculty explanations and demonstrations received a mean rating of 4.94 ± 0.24 (95% CI 4.82–5.07), with a median of 5 (IQR 5–5). The perceived relevance of the course to students’ educational needs as future physicians was rated 4.76 ± 0.44 (95% CI 4.54–4.99), with a median of 5 (IQR 5–5).

Interest in further exploring neonatal neurology and related topics during future training received a mean score of 4.29 ± 0.92 (95% CI 3.82–4.77), with a median of 5 (IQR 4–5).

The assessment of workload relative to the available course time yielded a mean score of 4.18 ± 1.19 (95% CI 3.57–4.79) and a median of 5 (IQR 3–5). Ten participants (58.8%) assigned a rating of 5, two (11.8%) a rating of 4, four (23.5%) a rating of 3, and one (5.9%) a rating of 1. The overall course evaluation and the workload item therefore showed the greatest variability among the evaluated items, despite both having a median rating of 5.

### 3.3. Evaluation of the Theoretical and Practical Components

The practical component received a mean rating of 4.88 ± 0.33 (95% CI 4.71–5.05), with a median of 5 (IQR 5–5). Fifteen participants (88.2%) assigned the highest rating of 5 and two (11.8%) assigned a rating of 4. In comparison, the theoretical component received a mean rating of 4.47 ± 0.62 (95% CI 4.15–4.79), with a median of 5 (IQR 4–5). Nine participants (52.9%) assigned a rating of 5, seven (41.2%) a rating of 4, and one (5.9%) a rating of 3. In an exploratory paired comparison, ratings of the practical component were higher than those of the theoretical component (Wilcoxon signed-rank test, *p* = 0.020). This comparison reflects participants’ perceived relevance of the two course components and should not be interpreted as evidence of superior learning associated with the practical component.

### 3.4. Self-Reported Knowledge

The retrospective pre-course knowledge self-rating had a mean score of 3.29 ± 1.10 (95% CI 2.73–3.86), with a median of 3 (IQR 2–4). Five participants (29.4%) assigned a rating of 2, five (29.4%) a rating of 3, four (23.5%) a rating of 4, and three (17.6%) a rating of 5.

The post-course knowledge self-rating had a mean score of 4.12 ± 0.70 (95% CI 3.76–4.48), with a median of 4 (IQR 4–5). Three participants (17.6%) assigned a rating of 3, nine (52.9%) a rating of 4, and five (29.4%) a rating of 5.

In an exploratory paired comparison, post-course self-ratings were higher than retrospective pre-course self-ratings (Wilcoxon signed-rank test, *p* = 0.010). However, both ratings were collected after completion of the course, with the pre-course rating based on retrospective self-assessment. This comparison therefore reflects a difference between participants’ post-course perception of their current knowledge and their retrospective perception of their pre-course knowledge and should not be interpreted as a prospective or objective measure of knowledge gain.

### 3.5. Transferability and Perceived Competence Acquisition

All 17 participants considered the educational format applicable to other areas of the medical curriculum (100%; 95% CI 81.6–100.0).

When asked whether the field-based educational experience had provided them with an initially useful professional competence, fourteen students stated that they had acquired at least an initial professionally useful competence (82.4%; 95% CI 59.0–93.8), while the remaining three selected “more yes than no”. These response categories were retained separately in Table 3.

Sixteen students considered the course format, duration, and allocated spaces consistent with their expectations (94.1%; 95% CI 73.0–99.0), while one participant was uncertain.

These responses describe participants’ perceptions of the applicability and professional relevance of the educational experience. They were not accompanied by an objective assessment of clinical or ultrasound competence or by an evaluation of subsequent implementation in clinical practice. Thus, all participants provided an overall favorable response regarding perceived competence acquisition (100%; 95% CI 81.6–100.0).

### 3.6. Expectations, Specialty Interest, and Curricular Replication

Seven respondents (41.2%) reported that the course met their expectations, whereas ten (58.8%) reported that it exceeded them. Regarding the possible influence of the course on considering pediatrics or neonatology as a future specialty, eight participants (47.1%) selected “yes”, seven (41.2%) selected “more yes than no”, and two (11.8%) selected “more no than yes”; none selected an unequivocally negative response. Overall, 15 of 17 participants (88.2%) therefore selected one of the two positive response categories.

These findings should be interpreted in the context of participant selection. Students voluntarily enrolled in the course and had an expressed interest in pediatrics and neonatal medicine, while baseline specialty interest was not formally quantified using a pre-course assessment. The responses therefore represent participants’ perceived post-course influence on their consideration of pediatrics or neonatology rather than a measured change in specialty preference. Fourteen respondents (82.4%) supported repeating the initiative and extending this educational approach to the core medical curriculum, three (17.6%) selected “do not know”, and none opposed its replication.

### 3.7. Free-Text Feedback

Eight of the 17 respondents (47.1%) provided at least one optional free-text response across the questionnaire. Because these responses were brief and were not collected or analyzed according to a predefined qualitative methodology, they are reported descriptively rather than as formal qualitative findings.

Positive comments highlighted the integration of theoretical and practical teaching, the opportunity to participate in small-group activities, and the perceived value of applying theoretical concepts within a practical clinical context.

Among suggestions for improvement, three respondents proposed closer temporal integration of the theoretical and practical activities, including the possibility of delivering them within a single day. Two respondents suggested greater direct involvement in cranial ultrasound scanning or additional opportunities for hands-on practice. Individual comments also suggested more detailed theoretical coverage of cranial ultrasound, inclusion of basic neonatal intensive care management topics, and discussion of a common neonatal condition that could subsequently be encountered in the clinical setting.

No formal thematic coding, investigator triangulation, or other qualitative analytic procedure was performed. The free-text responses are therefore presented solely as descriptive feedback and should not be interpreted as formal qualitative findings.

## 4. Discussion

This pilot evaluation suggests that a two-day educational activity integrated theoretical teaching with supervised practical exposure is highly acceptable to medical students.

Participants reported high overall satisfaction and curricular relevance, while the practical component received significantly higher ratings than the theoretical component.

Post-course knowledge self-ratings were also higher than retrospective ratings of pre-course knowledge. Furthermore, all respondents considered the educational format transferable to other curricular areas, and most supported its future inclusion in the medical curriculum. These findings describe learners’ immediate reactions and self-perceptions following the course. However, they should be interpreted as evidence of learner response and self-perceived learning rather than as proof of objective knowledge acquisition or clinical competence.

The course was developed in the context of broader concerns regarding undergraduate neurology education. Neurology is often perceived as difficult or insufficiently integrated with clinical practice, and neurophobia, concept originally described by Jozefowicz in 1994 [3], has been reported among medical students and early-career doctors [3,7,8]. The present questionnaire did not directly measure neurophobia and should therefore not be presented as an intervention proven to reduce it. Nevertheless, the favorable post-course ratings support the acceptability of the teaching format, linking theoretical foundations with bedside practice, but do not demonstrate a change in students’ attitudes toward or perceived approachability of neonatal neurology.

Participants’ higher ratings of the practical component are consistent with the appeal of experiential, clinically contextualized teaching in pediatric, neonatal, and ultrasound education [5,9,10,11]. However, this finding should also be considered in light of the characteristics of the participants and the course. Enrollment was voluntary, and participants had expressed an interest in pediatrics and, particularly, neonatal medicine. Moreover, none had previous experience with neonatal cranial ultrasonography. The novelty of the technique, the small-group format, direct interaction with faculty, the clinical environment, and the opportunity to handle the ultrasound probe under supervision may therefore all have contributed to the favorable ratings of the practical component. The comparison between theoretical and practical components should consequently be interpreted as a difference in perceived relevance rather than as evidence that the practical component resulted in greater learning.

The free-text responses provide additional descriptive information regarding participants’ perceptions of the course. Positive comments highlighted the integration of theoretical and practical teaching, small-group activities, and the opportunity to apply theoretical concepts within a clinical context. Suggestions for future editions included closer temporal integration of theoretical and practical activities and additional opportunities for direct involvement in cranial ultrasound scanning. These observations point to practical considerations for future course design, particularly the balance between observation and supervised student activity. Possible developments could include structured image-recognition exercises, standardized introductory checklists, simulation, anonymized image or case libraries, or additional carefully supervised probe-handling opportunities when clinically and ethically appropriate. These should be regarded as proposals for future course development rather than interventions evaluated in the present study.

Published educational work on neonatal cranial ultrasound has primarily focused on pediatric or neonatal postgraduate trainees. For example, Ben Fadel and McAleer reported objective improvements in neonatal cranial ultrasound knowledge and image interpretation performance following a structured web-based module for trainees [12]. More broadly, neonatal point-of-care ultrasound literature has emphasized the need for formal training, competency assessment, supervision, credentialing, and quality assurance [4,13]. Such standards are intended for clinicians who perform and interpret ultra-sound in practice and should not be interpreted as evidence that a brief undergraduate educational experience can confer scanning competence. At the undergraduate level, more appropriate objectives include introductory image literacy, familiarity with basic probe handling and standard scanning planes, awareness of the clinical role and limitations of cranial ultrasound and understanding of how imaging findings are integrated with perinatal history and neurological assessment. In the present course, students’ supervised probe handling was therefore intended as an introductory educational exposure rather than training toward independent scanning competence.

All respondents considered the educational format potentially applicable to other areas of the medical curriculum, and most supported repeating the initiative and extending this educational approach to the core curriculum. These perceptions can be considered in the broader context of literature on early clinical experience, which describes opportunities to connect classroom learning with clinical settings [14]. However, the present survey did not assess the mechanisms underlying these perceptions or demonstrate transferability. The responses indicate perceived applicability only; the course was not evaluated in another curricular area, institution, or learner population. Formal feasibility was likewise not assessed. Faculty time, equipment availability, clinical space, patient availability, supervision requirements, consent procedures, and potential effects on routine clinical care would need to be prospectively evaluated before broader implementation. The detailed description of the intervention provided in this report is intended to improve transparency and potential reproducibility, rather than to establish that the course can be implemented elsewhere without adaptation.

The specialty-interest findings also require cautious interpretation. Fifteen of 17 participants selected one of the two positive response categories when asked whether the course had influenced their consideration of pediatrics or neonatology as a possible future specialty. Specialty choice, however, is multifactorial and may change throughout medical training [15]. In addition, students voluntarily enrolled in the course and had already expressed an interest in pediatrics and neonatal medicine, while baseline specialty interest was not formally quantified using a pre-course assessment. The questionnaire therefore captured only participants’ immediate post-course perception of possible influence. These findings cannot demonstrate an increase in specialty interest or predict subsequent electives, research activity, postgraduate training, or career choice.

This study has several limitations. It was conducted at a single institution and included only 17 participants. Although all course attendees completed the questionnaire, resulting in a response rate of 100% (17/17), course enrollment itself was voluntary. Consequently, the complete questionnaire response rate does not eliminate the substantial potential for self-selection bias. Participants were medical students in the later years of their degree program (five fourth-year, six fifth-year, and six sixth-year students), were predominantly female, and had an expressed interest in pediatrics and neonatal medicine. None had previous experience with neonatal cranial ultrasonography. These characteristics limit the generalizability of the findings to students with different levels of training, prior ultrasound exposure, or specialty interests. In addition, ceiling effects were evident for several questionnaire items, limiting discrimination among highly favorable responses.

The questionnaire was developed locally for this educational initiative and was not psychometrically validated. Both knowledge ratings were obtained after completion of the course: participants rated their current knowledge and retrospectively rated their knowledge before attending the course. Retrospective pre-course ratings may be influenced by recall bias, social desirability, and changes in respondents’ internal standards [16,17]. The observed difference between the two ratings should therefore be interpreted as a difference in perceived knowledge rather than as evidence of actual knowledge acquisition. Providing the complete questionnaire as Supplementary Material improves transparency regarding item wording and response options but does not eliminate potential measurement or response bias.

No comparator group, prospective baseline assessment, objective knowledge test, image-interpretation assessment, or structured evaluation of scanning technique was included. Although students were allowed to handle the ultrasound probe under direct supervision and attempt to reproduce the principal coronal and sagittal views, no objective or checklist-based assessment of their performance was conducted. The relative contributions of theoretical content, practical exposure, novelty, small-group teaching, faculty interaction, and the clinical environment therefore cannot be separated. The study also did not prospectively measure implementation barriers or resource use, and the optional free-text comments were not subjected to formal qualitative analysis. Finally, the paired statistical comparisons were exploratory and should not be interpreted as estimates of educational effect.

The contribution of this report is therefore primarily descriptive. It provides a detailed account of a short undergraduate course integrating neonatal neurology and cranial ultrasound and documents the immediate perceptions of a small group of participating medical students. Future studies should incorporate prospective baseline measurements, objective assessments of knowledge and image interpretation, validated or carefully piloted evaluation instruments, delayed follow-up, and larger and more diverse samples. Where appropriate, comparison of theory-only and integrated theory-and-practice formats could help determine whether practical exposure provides measurable additional educational value. Multicenter studies would also be needed to assess feasibility, reproducibility, and implementation across different educational and clinical settings.

## 5. Conclusions

In this pilot descriptive post-course evaluation, 17 self-selected fourth- to sixth-year medical students reported favorable perceptions of a two-day course integrating neonatal neurology and cranial ultrasound, with the practical component receiving particularly high ratings. Post-course knowledge self-ratings were higher than retrospective ratings of pre-course knowledge. However, because both ratings were obtained after the course and no prospective baseline or objective assessment of knowledge or performance was conducted, these findings support the acceptability and perceived educational value of the course but do not establish objective learning, procedural competence, or educational effectiveness.

The course provides a model for introducing senior medical students to the clinical role of neonatal cranial ultrasound within a broader framework of neonatal neurological assessment and clinical reasoning. Its feasibility and transferability to other learner groups, curricula, or institutions remain to be established. Future studies should include larger and more diverse samples, prospective and objective learning outcomes, and follow-up beyond the immediate completion of the course.

## Figures and Tables

**Table 1:** Course timetable, content, learning objectives, and delivery methods.

Component	Content	Intended Learning Objectives	Format, Duration, and Resources
Day 1: theoretical session	Neurodevelopment; brain vulnerability and neurological complications of prematurity; neurological disorders of the term neonate; principles of neonatal neuroimaging; main anatomical structures visualized on standard coronal and sagittal cranial ultrasound planes; principal pathological findings identifiable by cranial ultrasound; role of magnetic resonance imaging as a complementary diagnostic modality.	To provide students with an introductory understanding of neonatal brain development and neurological disorders, and to introduce the clinical role and potential of cranial ultrasound in the evaluation of neonatal neurological conditions. Students were expected to become familiar with the main anatomical structures visualized on standard ultrasound planes and with selected pathological findings.	7.5 h of theoretical teaching (4.5 h in the morning and 3 h in the afternoon). Frontal lectures sup-ported by PowerPoint presentations, illustrative ultrasound im-ages, and educational videos. Teaching was delivered by an experienced neonatologist from the Neonatal Pathology Unit of the Istituto Giannina Gaslini.
Day 2: neonatal neurological assessment	Structured overview of the neonatal neurological examination, including the main components of the examination and clinical examples.	To familiarize students with the main elements of the neonatal neurological examination and to understand the relationship between neurological clinical findings and the potential need for further diagnostic evaluation.	Included within the 5 h morning session. The neurological examination was presented through video demonstrations and explanatory teaching. Students observed the videos and discussion; the complete neurological examination was not performed directly on a neonate by the students.
Day 2: cranial ultrasound	Introduction to the use of a microconvex transducer through the anterior fontanelle; standard coronal and sagittal imaging planes; recognition of the main anatomical structures; selected pathological findings demonstrated through images and videos; bedside ultrasound ex-amination in three healthy term neonates.	To understand the clinical indications and potential applications of neonatal cranial ultrasound; to become familiar with probe handling and standard coronal and sagittal imaging planes; and to recognize the main anatomical structures previously presented during the theoretical session. The activity was not intended to establish independent scanning competence.	Supervised hands-on activity in small groups. The 17 students were divided into three groups (6, 6, and 5 students), each supervised by one neonatologist tutor. Ultrasound examinations were initially performed by the tutor, after which each student had the opportunity to handle the transducer under direct supervision and attempt to reproduce the demonstrated planes. Tutor-to-student ratios were 1:6 and 1:5.
Day 2: clinical integration	Integration of perinatal and neonatal his-tory, neurological clinical findings, and cranial ultrasound findings during the practical activity.	To help students understand when cranial ultrasound may be clinically useful based on the clinical presentation and relevant history, and to appreciate the relationship between clinical findings and ultrasound imaging.	No separate time slot was allocated to this activity. Clinical integration was incorporated throughout the supervised hands-on ultra-sound sessions, with tutors providing explanations and discussing the clinical relevance of the findings while reviewing and interpreting the ultrasound scans with the students.

**Table 2:** Descriptive results of the post-course questionnaire administered to participating medical students (n = 17). Data are presented as mean ± standard deviation (SD), 95% confidence interval (CI), median (interquartile range [IQR]), and n (%). Likert-scale items ranged from 1 (lowest rating) to 5 (highest rating). Percentages may not sum to 100% because of rounding. Confidence intervals were not constrained to the theoretical limits of the Likert scale.

Questionnaire Item	Mean ± SD	95% CI	Median (IQR)	1, n (%)	2, n (%)	3, n (%)	4, n (%)	5, n (%)
Overall course evaluation	4.18 ± 1.19	3.57–4.79	5 (3–5)	1 (5.9)	0	4 (23.5)	2 (11.8)	10 (58.8)
Alignment with the existing medical curriculum	4.94 ± 0.24	4.82–5.07	5 (5–5)	0	0	0	1 (5.9)	16 (94.1)
Retrospective pre-course knowledge self-rating ^2^	3.29 ± 1.10	2.73–3.86	3 (2–4)	0	5 (29.4)	5 (29.4)	4 (23.5)	3 (17.6)
Interest generated by the course topics	4.65 ± 0.61	4.34–4.96	5 (4–5)	0	0	1 (5.9)	4 (23.5)	12 (70.6)
Relevance of theoretical component ^1^	4.47 ± 0.62	4.15–4.79	5 (4–5)	0	0	1 (5.9)	7 (41.2)	9 (52.9)
Clarity of topics presented	4.76 ± 0.56	4.48–5.05	5 (5–5)	0	0	1 (5.9)	2 (11.8)	14 (82.4)
Workload relative to time available	4.18 ± 1.19	3.57–4.79	5 (3–5)	1 (5.9)	0	4 (23.5)	2 (11.8)	10 (58.8)
Relevance of practical component ^1^	4.88 ± 0.33	4.71–5.05	5 (5–5)	0	0	0	2 (11.8)	15 (88.2)
Relevance to education as future physicians	4.76 ± 0.44	4.54–4.99	5 (5–5)	0	0	0	4 (23.5)	13 (76.5)
Clarity of faculty explanations/demonstrations	4.94 ± 0.24	4.82–5.07	5 (5–5)	0	0	0	1 (5.9)	16 (94.1)
Interest in further exploring the topics	4.29 ± 0.92	3.82–4.77	5 (4–5)	0	1 (5.9)	2 (11.8)	5 (29.4)	9 (52.9)
Post-course knowledge self-rating ^2^	4.12 ± 0.70	3.76–4.48	4 (4–5)	0	0	3 (17.6)	9 (52.9)	5 (29.4)

^1^ Exploratory paired comparison between the practical and theoretical components using the Wilcoxon signed-rank test: *p* = 0.020. The comparison reflects participants’ perceived relevance of the two components and should not be interpreted as evidence of superior learning associated with the practical component. ^2^ Both knowledge ratings were obtained after completion of the course; the pre-course rating was retrospective. Exploratory paired comparison using the Wilcoxon signed-rank test: *p* = 0.010. This comparison should not be interpreted as a prospective or objective measure of knowledge gain.

**Table 3:** Categorical questionnaire items.

Questionnaire Item	Response Category	n (%)
Applicability of this educational format to other areas of the medical curriculum	Yes	17 (100)
	No/other responses	0
Perceived acquisition of an introductory professionally relevant competence	Yes	14 (82.4)
	More yes than no	3 (17.6)
Educational format, duration, and facilities consistent with expectations	Yes	16 (94.1)
	Don’t know	1 (5.9)
Course compared with participants’ expectations	Exceeded expectations	10 (58.8)
	Met expectations	7 (41.2)
Course influence on considering pediatrics/neonatology as a future specialty ^1^	Yes	8 (47.1)
	More yes than no	7 (41.2)
	More no than yes	2 (11.8)
	No	0
Support for repeating the initiative and extending it to the core curriculum	Yes	14 (82.4)
	Don’t know	3 (17.6)
	No	0

Data are presented as n (%). Original questionnaire response categories are retained where applicable. Percentages may not sum to 100% because of rounding. ^1^ Baseline specialty interest was not formally measured using a pre-course assessment. Participants voluntarily enrolled in the course and had an expressed interest in pediatrics and neonatal medicine; therefore, responses to this item represent perceived post-course influence rather than a measured change in specialty preference.

## Data Availability

The data are not publicly available due to privacy or ethical restrictions. The data presented in this study are available on request from the corresponding author.

## Supplementary Materials

The following supporting information can be downloaded at: https://www.mdpi.com/article/10.3390/pediatric18050124/s1, File S1: English-language version of the post-course questionnaire.
