# Supplementary material for: Early Exposure to Neonatal Neurology and Cranial Ultrasound: A Pilot Evaluation of an Undergraduate Course
Source: Pediatr Rep. 2026 Sep 22;18(5):124. doi: 10.3390/pediatric18050124 (PMC13708407; doi:10.3390/pediatric18050124)
Supplement: Supplementary file 1 [file pediatrrep-18-00124-s001.zip › pediatrrep-4468078-supplementary.pdf]

Supplementary Material File S1
English-language version of the post-course questionnaire

Note. This document is an English translation of the questionnaire originally administered in Italian. The order, response options, and meaning of the original items have been preserved. ADE refers to the elective educational activity through which the course was offered.

Dear Students,
We kindly ask you to complete this brief satisfaction survey regarding the two-day monographic course you attended in the Neonatal Intensive Care Unit as an ADE.

* Required item

1. Year of medical school *

○ First
○ Second
○ Third
○ Fourth
○ Fifth
○ Sixth

2. Sex *

○ Male
○ Female
○ Prefer not to specify

3. How would you rate the initiative overall? *

1	2	3	4	5

4. How would you rate the overall alignment of the topics covered with your current medical curriculum? *

1	2	3	4	5

5. How would you rate your knowledge of the topics covered BEFORE attending the ADE course? *

1	2	3	4	5

6. How would you rate the topics covered overall in relation to your personal interests and professional inclinations? *

1	2	3	4	5

Day 1

7. How would you rate the relevance of the topics covered during the theoretical session (neurodevelopment and neonatal neurological disorders)? *

1	2	3	4	5

8. How would you rate the clarity of the topics presented? *

1	2	3	4	5

9. How would you rate the workload relative to the time available? *

1	2	3	4	5

10. Were there any topics, whether covered or not covered, that you would have liked to explore in greater depth or have included?

Day 2

11. How would you rate the relevance of the topics covered during the practical session (neonatal neurological examination and cranial ultrasound)? *

1	2	3	4	5

12. How would you rate the relevance of the topics covered to your educational needs as a physician in training? *

1	2	3	4	5

13. Do you think that this educational format (a monographic course with a practical demonstration) could be applied to areas other than your current training pathway? *

○ Yes
○ No
○ More no than yes
○ More yes than no
○ I do not know

14. How would you rate the clarity of the explanations and demonstrations provided by the instructors? *

1	2	3	4	5

15. Do you think that this field-based training provided you with a professionally useful competence, even at an introductory level? *

○ Yes
○ No
○ More yes than no
○ More no than yes
○ I do not know

16. To what extent would you like to explore the topics covered further during your future training? *

1	2	3	4	5

17. Do you think that the educational format, duration, and teaching spaces were consistent with your expectations? *

○ Yes
○ No
○ I do not know

18. Were there any topics, whether covered or not covered, that you would have liked to explore in greater depth or have included?

Final considerations

19. How would you rate your knowledge of the topics covered AFTER attending the ADE course? *

1	2	3	4	5

20. Did attending the monographic course influence your consideration of pediatrics/neonatology as a possible choice for your future specialty training? *

○ Yes
○ No
○ More yes than no
○ More no than yes
○ I do not know

21. Based on your academic experience, did the course meet your expectations? *

○ It exceeded my expectations
○ It was what I expected
○ I expected something more
○ It did not meet any of my expectations

22. Do you think that this initiative should be offered again in the future and extended to all students as part of the core curriculum? *

○ Yes
○ No
○ I do not know

23. Do you have any comments for us?

